# Very Long-Term Outcome of Non-Surgically Treated Patients with Temporal Lobe Epilepsy with Hippocampal Sclerosis: A Retrospective Study

**DOI:** 10.1371/journal.pone.0159464

**Published:** 2016-07-14

**Authors:** Tsugiko Kurita, Kotaro Sakurai, Youji Takeda, Toru Horinouchi, Ichiro Kusumi

**Affiliations:** Department of Psychiatry and Neurology, Hokkaido University Graduate School of Medicine, Sapporo, Japan; Cleveland Clinic, UNITED STATES

## Abstract

**Objective:**

Surgical intervention can result in complete seizure remission rates of up to 80% in patients with temporal lobe epilepsy with hippocampal sclerosis (TLE-HS). However, certain patients cannot be treated surgically for various reasons. We analyzed the very long-term clinical outcomes of patients with TLE-HS who could not be treated surgically.

**Methods:**

Subjects were selected from among patients with TLE-HS who were actively followed up for >10 years and treated with medication without surgical treatment. Patient medical records were used to retrospectively study seizure frequency, various clinical factors, and social adjustment. Patients who were seizure-free or had only aura were classified into Group 1; the others were classified into Group 2. Clinical factors including both patient and disease-specific factors were compared between the two groups. Current social adjustment, including the education, work, and economic status of each patient, was also investigated.

**Results:**

Forty-one (41) subjects met the criteria for analysis, of which 12 (29%) were classified into Group 1. The average age of patients in Group 1 was higher than that of Group 2 (p = 0.0468). Group 2 included a significantly higher rate of patients who had more than one seizure per week at the onset (p = 0.0328), as well as a greater mean number of anti-epileptic drugs taken (p = 0.0024). Regarding social adjustment, Group 2 contained significantly fewer current jobholders than Group 1 (p = 0.0288).

**Conclusions:**

After very long-term follow-up periods, 29% of patients with TLE-HS had a good outcome through treatment with anticonvulsant medications. Older patients tended to have fewer seizures, and seizure frequency at the onset was the only factor that predicted outcome.

## Introduction

Temporal lobe epilepsy with hippocampal sclerosis (TLE-HS), the most frequent epilepsy syndrome, is generally refractory to anti-epileptic drugs. Epilepsy surgeries, such as anterior temporal lobectomy or selective amygdalo-hippocampectomy, provide a complete seizure remission rate of up to 60–80% in TLE-HS [1–8]. A randomized, controlled trial of epilepsy surgery for patients with TLE-HS demonstrated a better outcome after surgery versus medical treatment [2,8]; indeed, the standard treatment plan for TLE-HS without seizure control by medication is surgical resection [9]. However, some patients with TLE-HS still do not undergo surgery for various reasons, including medical (bilateral focus, psychiatric symptoms) or economic reasons, or sometimes simply out of respect for the patient’s wishes. Previous studies assessing the prognosis for seizure control by medication in patients with TLE-HS are limited to relatively short-term follow-ups of 1–2 years [2,8,10]. Only a few studies have reported the long-term outcomes in these patients [3,11], and outcomes for durations more than a decade are not clear. Moreover, since the subjects of the cited studies were candidates for surgical treatment, these studies were biased towards refractory cases with relatively poor prognoses.

The purpose of this study was to investigate the very long-term (> 10 years) outcome in cases of non-surgical treatment for TLE-HS, and to identify predictors for successful seizure control in such cases.

## Materials and Methods

This retrospective study was approved by the independent ethics committee of Hokkaido University Hospital. Subjects were selected among the database of 1781 patients with epilepsy at the Department of Psychiatry and Neurology, Hokkaido University Hospital, between 1947 and 2011. We reviewed the medical records of patients who met the following inclusion criteria: a) patients actively followed-up over 10 years by epileptologists at our hospital; b) medical history and seizure semiology was consistent with that of temporal lobe epilepsy (e.g. simple partial seizures of the déjà-vu or jamais-vu type; or including epigastric or psychic manifestations, followed by complex partial seizures characterized by staring and oral automatisms with or without superior limb automatisms or contralateral superior limb dystonia); c) HS was evident on MRI as signal hyperintensity within the hippocampus on T2-weighted or fluid-attenuated inversion-recovery (FLAIR) images, or as hippocampal atrophy on coronal T1-weighted images; d) patients who had not undergone surgical resection.

Patients were excluded based on the following criteria: a) signs of non-temporal lobe origin, such as visual aura, simple motor, or simple sensory aura; and b) ictal or interictal encephalography, SPECT, or PET imaging that were contradictory to TLE.

MRI examinations were performed using a 1.5-Tesla scanner (MR Systems Achieva, Philips). MRI examinations included axial and coronal slices of T1-weighted images, T2-weighted images, and FLAIR images, in order to optimize the visualization of the mesial temporal structures.

The inclusion criteria for HS were: a) hippocampal atrophy observed on T1-weighted images, b) increased mesial temporal signal intensity alteration on T2-weighted images and FLAIR images, and c) disruption of the internal hippocampal architecture on T1-weighted images.

Expert epileptologists and neuroradiologists reviewed the MRI scans of each patient independently. Each observer was asked to make an overall diagnosis and to confirm the lateralization of the lesion. Cases for this study were chosen if HS was diagnosed independently by at least two observers. Patients suspected to have other abnormal findings such as focal cortical dysplasia, tumor, or cerebral infarction were excluded.

Each patient had been evaluated electroencephalographically using the routine international 10/20 recording system. We considered that ictal rhythmic discharges, interictal spikes, or sharp waves around the temporal area were consistent with TLE.

We investigated seizure frequency in the last 2 years and divided the patients into two groups. Patients who were seizure free or had only aura were classified into Group 1. Patients who had complex partial seizures (CPS) or secondary generalized tonic-clonic seizures (GTCs) within the time period were classified to Group 2. Additionally, medical records were used to verify the length of time between the last seizure and trigger event and the control of seizures in Group 1, as well as the transition of seizure frequency during follow-up periods in Group 2. Clinical factors such as sex, age, age of onset, durations of epilepsy, side of HS in MRI, presence of febrile seizures, GTCs, and the numbers of anti-epileptic drugs (AEDs) taken before were extracted from the medical records. Epileptic psychosis was defined as the presence of psychosis with hallucination-delusion, agitation, or aggression (so-called “schizophrenia-like” symptoms) during treatment for epilepsy. Seizure frequency at the onset (before starting anti-epileptic drug treatment) was also extracted and classified as having a frequency of ≥1/week. The reason given for the selection of non-surgical treatment was also documented.

Furthermore, we investigated social adjustment via the following indicators: Final degree of education, employment status, marital history, and having children (for women only). We compared the clinical factors and social adjustment indicators between the two groups.

Student’s t-test was used to statistically analyze the group means of age, age of onset, duration of epilepsy, and the number of AEDs. Fisher’s exact test (two-tailed) was used to compare sex, side of HS, febrile seizures, GTCs, seizure frequency at the onset, the number of patients with epileptic psychosis, and the number who were surgical candidates after presurgical evaluation. The level for statistical significance was set at P < 0.05. Patient records/information were anonymized and de-identified prior to analysis.

## Results

Forty-one patients (13 men, 28 women; average age 53.1±12.5 years) met the inclusion criteria for our study. The mean follow-up period in our hospital was 27.3±13.0 years. The average age of epilepsy onset was 12.2±11.0 years, and the average duration of epilepsy was 40.4±12.3 years. Seventeen patients (41%) had left side HS, and 22 (54%) had right side HS; the remaining 2 patients had bilateral HS. Twenty-one patients (51%) had experienced febrile convulsions. Thirty patients (73%) had experienced generalized tonic-clonic seizures more than once. The patients with uncontrolled seizures had been informed about surgical resection as a treatment option by the physician, but for various reasons they had refused the recommendation.

**Fig 1:**
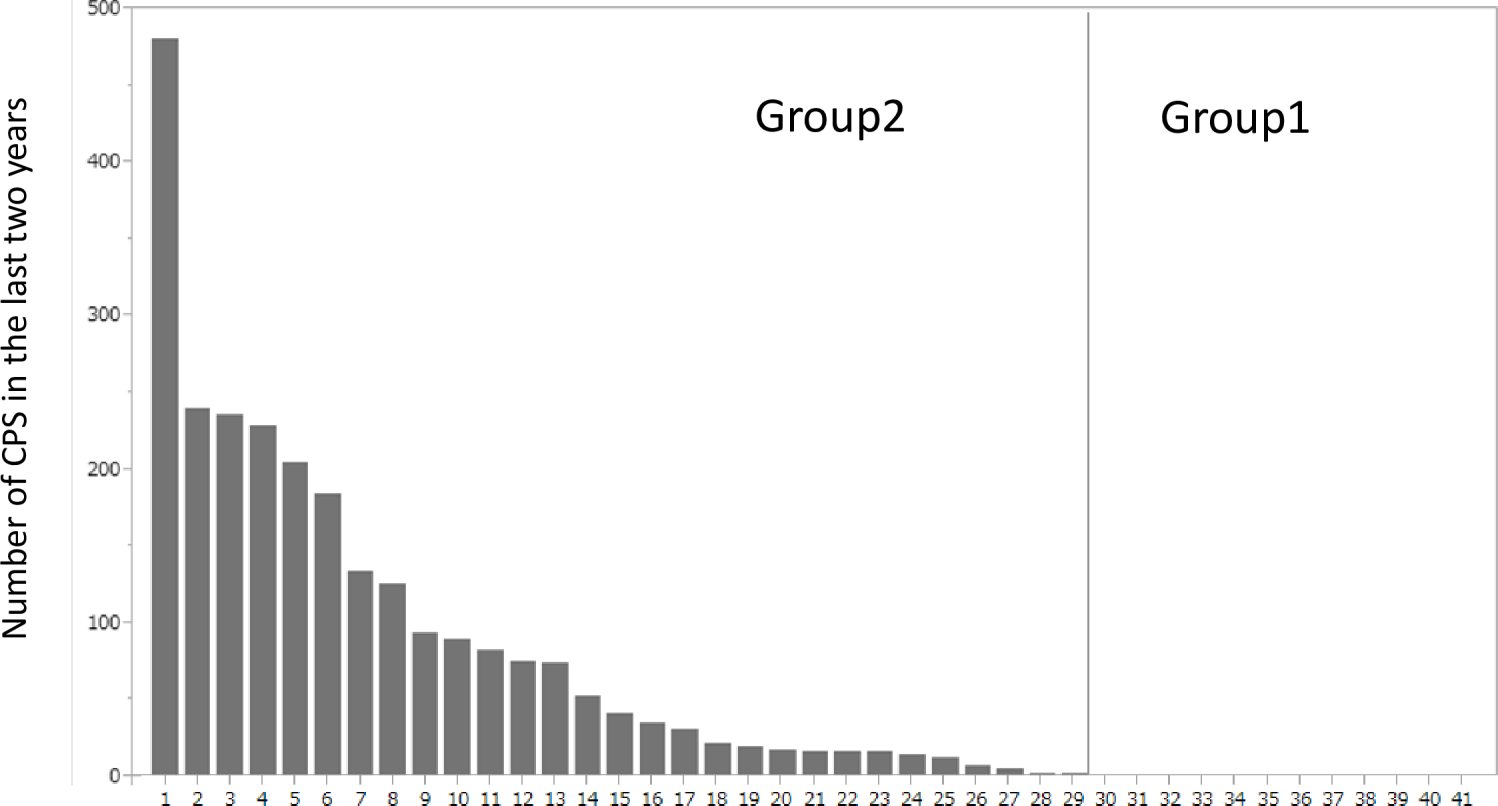
Frequency of complex partial seizures (CPS) in the patient groups. Patients were divided into Group 1 (less than 1 seizure/week at onset) and Group 2 (greater than or equal to 1/week). The frequency of CPS in each group over the 2-year assessment period is provided.

Fig 1 demonstrates the seizure frequency over the last 2 years. Group 1, whose TLE was rated as relatively less severe, consisted of 12 patients (29%).Group 2 consisted of 29 patients (71%). In Group 2, 12 of the 29 patients had seizures less than once per month, while the remaining 17 patients had more frequent seizures.

The clinical characteristics of the patients in each group are summarized in Table 1. The average age of patients in Group 1 was older than Group 2 (p = 0.0468). Sex, duration of epilepsy, age of onset, laterality of HS, febrile seizures, and presence of GTCs were not significantly different between the groups. Significantly more patients in Group 2 suffered from 1 or more seizures per week at the onset (p = 0.0328). Though more patients in Group 2 had been diagnosed with epileptic psychosis, the difference was not statistically significant. Many patients refused even the presurgical evaluation; therefore, only 7 patients in Group 2 were identified as surgical candidates. The most common reason for the selection of non-surgical treatment was refusal of surgery (24 patients). Other patients excluded from surgical treatment included: Six patients reporting economic reasons, 6 other patients with only rare or simple partial seizures, 4 further patients with psychiatric problems, and one with bilateral foci (see S1 Table for details). Of the physical characteristics, the only other significant difference between groups was in the mean number of AEDs taken before this study. Patients of Group 2 had taken a mean of 5.5±1.9 AEDs, versus 3.5±1.6 AEDs in Group 1 (p = 0.0024). The means of AEDs taken at the time of investigation were 1.3±0.9 for Group 1 and 2.1±0.8 for Group 2 (p = 0.0083).

**Table 1:** Clinical characteristics of the study subjects by group.

	Group 1 *(n = 12)*	Group 2 *(n = 29)*	P value
**Sex**	4 male, 8 female	9 male, 20 female	1.0000
**Age (years)**	59.2±15.0	50.5±11.0	0.0468*
**Onset age (years)**	13.0±13.5	11.8±10.0	0.7600
**Duration of epilepsy (years)**	45.3±16.1	38.4±10.0	0.1080
**Laterality of HS**	left 5, right 7	left 12, right 15, bilateral 2	1.0000
**Febrile seizures (n)**	5	16	0.5055
**Presence of sGTC (n)**	9	21	1.0000
**Seizure frequency at onset**			0.0328*
***≥1/week***	1	13	
***<1/week***	11	16	
**number of AEDs taken before**	3.5±1.6	5.5±1.9	0.0024*
**Epileptic psychosis**	1	9	0.2313
**Presurgical evaluation (n)**	0	7	0.0850

*P<0.05

Mean duration from the last seizure in Group 1 was 14.29 ±10.30 years, and the longest duration was 38 years. In 8 patients, no seizure had occurred in the last 10 years. The trigger event leading to seizure control was a change or increase in medication in 6 patients, first medication in 2 patients, a gradual reduction in 3 patients, and rare seizure from the onset in 1 patient. The transition of seizure frequency during follow-up periods in Group 2 was as follows: 12 patients had constant seizures, 8 patients experienced exacerbation after a seizure-free period, 7 patients had a gradual reduction in seizure frequency, and 2 patients had a progressively worsening course (see S1 Table for details).

Social adjustment values for each group are summarized in Table 2. Educational backgrounds were not different between groups; however, Group 2 had fewer jobholders than did Group 1 (p = 0.0288). Six patients in Group 2 (20.7%) were on public assistance, while no patients of Group 1 were on.

**Table 2:** Social adjustment values for study subjects by group.

	Group 1 (n = 12)	Group 2 (n = 29)	P value
**Education**			0.0847
*only compulsory education*	4 (33.3%)	9 (31%)	
*school of handicapped children*	0 (0%)	2 (6.9%)	
*high school*	7 (58.3%)	7 (24.1%)	
*junior or technical college*	0 (0%)	9 (31%)	
*university*	1 (8.3%)	2 (6.9%)	
**Jobholders**	7 (58.3%)	6 (20.7%)	0.0288*
**Without a regular occupation**	5 (41.7%)	23 (79.3%)	
*on public assistance*	0 (0%)	6 (20.7%)	
*homemakers*	3 (25%)	7 (24.1%)	
*others*	2 (16.7%)	10 (34.5%)	
**Marital history**	5(41.7%)	15 (51.7%)	0.7337
**Women having children**	2/8 (25%)	9/20 (45%)	0.4188

*P<0.05

## Discussion

TLE-HS, a medically intractable type of epilepsy, is the most common form of surgically remediable epileptic syndrome. Wiebe et al. conducted a randomized controlled trial assessing surgical intervention, finding that the cumulative proportion of patients who were free of seizures impairing awareness was 58 percent in the surgical group versus 8 percent in the medical group at 1 year [2]. They concluded that surgery was superior to medical therapy in TLE-HS. Therefore, surgical treatment such as anterior temporal lobectomy or selective amygdalo-hippocampectomy should be considered an option, at an adequate time after appropriate information has been provided to patients [9]. However, a significant number of patients with TLE-HS either cannot or will not have surgery, and instead continue anti-epileptic pharmacotherapy. Medical reasons to deny surgery include bilateral foci, undetermined laterality of epileptic seizures, or psychiatric complications. Furthermore, surgery may be deemed too invasive in cases when seizures are well-controlled medically, because of the risk of postoperative memory problems, especially after a left-side resection [2, 5, 12]. Postoperative complications, though relatively rare in TLE-HS surgeries, still include possible fatality (e.g., from unusual bleeding or infection) [13]. Other reasons can include economic burden, the lack of neurosurgeons with the necessary specialized training, and the patient’s beliefs (e.g. a negative predisposition toward brain surgery). Unlike malignant progressive lesions, patients adapted to living with epileptic seizures do not always desire brain surgery. The burdens of time, cost, and invasiveness for surgical treatment are higher than those for medication, contributing towards patients’ negative views of surgical treatment.

Only a few studies made reference to the long-term outcome in non-surgically treated populations with epilepsy. One study reported that the cumulative proportion of patients free of all seizures was 12% in the clinical group after a 1-year follow-up [7]. Another study showed that 21% of non-surgically treated patients with medically refractory, localization-related epilepsy were free from seizure after an average of 4.4 years from surgical evaluation [11]. Although their study included patients with other seizure disorders besides TLE, they concluded that the long-term prognosis in patients with refractory partial epilepsy who are not surgical candidates may be more positive than might be generally expected. The other retrospective study reported that in patients with TLE-HS using medication, 23.4% became seizure-free after a mean follow-up period of 3.4 years [3]. Twenty-five percent of patients with TLE-HS maintained their seizure-free status for 1 year after a 2-year follow-up, with medication only [10].

Our results, after an average of a 27.3-year follow-up period, showed a slightly higher rate relative to the above reports, such that 29% of patients were seizure-free. We hypothesize two reasons for this difference. The first possible factor was aging. The average of age in our study was significantly older than that in previous reports [10, 11]. Group 1 also included significantly older patients than Group 2, which suggests the possibility of a naturally progressing decline in seizure activity with age. Epidemiological studies have revealed that epilepsy is most common among elderly persons [14], but the impact of aging on the course of epilepsy is unknown. Some neuroimaging literature demonstrated morphometric changes in white matter, including the bilateral frontal lobes, bilateral temporal lobes, corpus callosum, and bilateral cerebellar hemispheres, in TLE-HS [15, 16]. On the other hand, brain imaging studies of normally aging people have revealed age-related volume reductions in the medial temporal lobes and prefrontal cortex [17]. There is no evident information addressing the alteration of brain structure and seizure frequency in aging patients with chronic TLE-HS. In Group 1, 3 patients experienced eventual seizure freedom for 2 years after a gradual reduction. In Group 2, 24.1% experienced a gradual reduction of seizures over the course of medical treatment, which was the most noteworthy transition in this group. On the other hand, only 8 patients in Group 1 achieved seizure freedom after 10 years, and 8 patients in Group 2 in fact experienced exacerbation after a period of being seizure-free. Therefore, we must conclude that constant vigilance regarding the risk of seizure recurrence is necessary.

The other factor is the selection of patients. The subjects of previous reports were refractory cases being assessed for surgical treatment [2,3,7,8,11]. Our study included patients other than those refractory cases, so a more natural improvement ratio could be obtained. One study demonstrated that 38.6% of sporadic benign temporal lobe epilepsy cases had MRI evidence of unilateral HS [18]. Surgical resection in these cases was not considered necessary.

The seizure frequency before medical treatment was considered the best prognostic factor in this study. More patients with at least weekly seizures were found in Group 2 than in Group 1. This result indicates that more seizure activity at onset was related to the difficulty of seizure control, a relationship that corresponded with the results of the preceding studies [19, 20]. This relationship represents a strong argument for the necessity of early medical treatment for TLE-HS.

Patients in Group 2 had been prescribed more AEDs in the past, and took more AEDs at the time of investigation, than patients in Group 1. Group 2 included more refractory cases, which likely explains the increased use of medication. This result fits well with those of a previous study, which reported that epileptic seizures in most cases were controlled by the first or second AED, and the possibility of full remission of seizure activity was significantly reduced after the third attempted AED [21]. Thus, in cases where an appropriate second AED has failed, the option of surgical treatment should be presented to patients as that with the best potential outcome.

Epilepsy has a marked negative impact on psychosocial outcomes compared with the general population, especially regarding marriage, having children, educational achievement, and work [22]. Thus, the social adjustment of the patients is a matter that demands careful consideration. Patients with at least a high school diploma were equally represented in both groups, though some slight differences were noticeable. In Group 1, 58.3% of the patients were working, while 79.3% of the patients in Group 2 could not hold a job. No patients in Group 1, but 20.7% of patients in Group 2, were on public assistance. Almost half of the patients with epilepsy undergoing treatment at a general hospital were reported to have a job [23]. In the cited study, most patients had uncomplicated epilepsies, and the authors did not investigate the relationship between holding a job and seizure outcomes. In patients who underwent surgery for refractory TLE, 56.9% were employed, and 75.2% had been seizure-free for a period of at least 1 year before the last follow up [12]. Although social adjustment was affected by various factors outside of seizure condition, the sudden loss of consciousness these patients could face with seizure disorders could unfortunately also restrict their choice of treatment in the face of possibly losing their jobs.

Our study was limited by its small sample size and the retrospective nature of the study. Now, however, the standard treatment plan for refractory TLE-HS is surgical resection, so performing a controlled trial of such a long follow-up duration regarding TLE-HS was impractical. Despite this limitation, we clarified the outcomes in a very long-term follow-up period for patients with TLE-HS who had not undergone surgical resection.

## Conclusions

In studying the outcomes of non-surgically treated patients with TLE-HS over an average follow-up period of almost 30 years, we found that 29% of the patients became seizure-free, though 54% still had seizures more than once a month even after lengthy AED medication. Our older patients tended to have fewer seizures, and the best prognostic indicator was the frequency of seizures at onset, such that a lower frequency led to a good outcome. This long-term prognosis could be helpful information to aid the decision of patients with TLE-HS who are hesitant to undergo surgical treatment.

## Supporting Information

S1 Table Detailed clinical data of the individual patient. (XLSX)
